# Frequency of Coinfection on the Vaginal Wet Preparation in the Emergency Department

**DOI:** 10.7759/cureus.11566

**Published:** 2020-11-19

**Authors:** Justin M Elkins, Santiago Cantillo-Campos, Johnathan M Sheele

**Affiliations:** 1 Emergency Medicine, Mayo Clinic, Jacksonville, USA

**Keywords:** bacterial vaginosis, candidiasis, coinfection, emergency department, trichomonas, vaginitis, emergency medicine, vaginal discharge, wet prep, vaginal wet preparation

## Abstract

Introduction

Vaginal infections are common in the emergency department (ED) but the frequency of vaginal coinfections identified on wet preparation is unknown.

Methods

The study examined a data set of 75,000 ED patient encounters between April 18, 2014, and March 7, 2017, who had received testing for gonorrhea, chlamydia, or trichomonas or had received a urinalysis and urine culture during the ED encounter. From this data set we reviewed 16,484 patient encounters where a vaginal wet preparation was performed on women age 18 years and older. Findings from the vaginal wet preparation and ED discharge diagnoses were examined to evaluate the frequency of vaginal coinfections with vulvovaginal candidiasis, trichomoniasis, and bacterial vaginosis.

Results

Among the women who had wet preparations, 4,124 patient encounters (25.0%) had a diagnosis of bacterial vaginosis, 625 (3.8%) had a diagnosis of vulvovaginal candidiasis, and 1,802 (10.9%) were infected with *Trichomonas vaginalis*. Twenty encounters (0.1%) had a diagnosis of vulvovaginal candidiasis and trichomoniasis; 150 (0.9%), bacterial vaginosis and trichomoniasis; 136 (0.8%), vulvovaginal candidiasis and bacterial vaginosis; and 10 (0.1%), trichomoniasis, bacterial vaginosis, and vulvovaginal candidiasis. On vaginal wet preparation, the mean white blood cell count was 13.0 per high-power field. Clue cells were found in 6,988 wet preparations (42.4%); 1,065 wet preparations (6.5%) had yeast and 1,377 (8.4%) had *T. vaginalis*. *T. vaginalis* was identified in 2.5% (266/10,542) of urinalyses and 8.4% (406/4,821) of nucleic acid amplification tests.

Conclusions

Vaginal coinfections were uncommon among women receiving a vaginal wet preparation in the emergency department. The most common vaginal coinfection was bacterial vaginosis and trichomonas.

## Introduction

Genitourinary concerns are common in the emergency department (ED), where high rates of sexually transmitted infections (STIs) are diagnosed [1]. Although pelvic examinations are routinely performed for women in the ED with genital concerns, the examination itself has questionable utility in helping to diagnose STIs [2-4]. However, pelvic examination and vaginal wet preparation (hereafter called wet prep) can be helpful for establishing a cause for vulvovaginal symptoms, which is an irritation or inflammatory condition of the vagina that can present with an associated odor, itching, and swelling [5,6]. Bacterial vaginosis (BV), vulvovaginal candidiasis, and *Trichomonas vaginalis* infection, in order of decreasing frequency, account for about 70% to 90% of vaginitis causes [5-8]. Other causes include desquamative inflammatory vaginitis, aerobic vaginitis, vaginal erosive disease, and atrophic vaginitis [5,6,9]. In outpatient clinics, BV is the most common cause of vaginitis, followed by vulvovaginal candidiasis and trichomonas; however, disease prevalence is likely different for an ED population [10,11].

Most wet preps in the ED are performed in women of reproductive age whose vaginal potential of hydrogen (pH) typically ranges from about 4.0 to 5.0 [6,12]. The normal acidic vaginal environment helps inhibit the growth of some pathogenic organisms [12]. A disruption in the normal vaginal microbiome can result in vaginitis from STIs, antibiotic therapy, contraception, douching, foreign bodies [12,13]. Vaginal pH greater than 4.5 can occur with BV or trichomoniasis; a pH of 4.0 to 4.5 can be seen with vulvovaginal candidiasis [12].

Vaginal coinfections have been reported in 5% to 30% of women in outpatient gynecology and STI clinics, but none of these studies examined an ED population [14-17]. The coinfection rates for BV and *T. vaginalis* have been reported to be up to 70%; for BV and vulvovaginal candidiasis, about 25% [14]. The objective of the present study was to determine the frequency of vaginal coinfections among women undergoing vaginal wet prep in the ED.

## Materials and methods

Institutional review board approval was obtained from University Hospitals (UH) and Mayo Clinic, and a waiver of informed consent was obtained. We examined a data set of 75,000 UH ED patient encounters that involved testing for gonorrhea, chlamydia, or trichomonas or who had a urinalysis and urine culture obtained in the ED. Data has been previously published from the data set used in this analysis [18]. The data set contained data obtained from the UH electronic health records by a non-clinician who was blinded to the specific study objectives. All patients in the data set were age 18 years or older, and had an ED visit between April 18, 2014, and March 7, 2017. We examined only the data of female patients who had an ED vaginal wet prep result. The vaginal wet preparation white blood cells (WBCs) were reported as ranges 0-5, 5-10, 11-15, 15-25, 25-50, and 50-100 cells per high-power field (HPF), and the means of these ranges were used in the analysis: 2.5, 7.5, 13, 20, 37.5, and 75 respectively so that the data could be modeled as a continuous variable. Vaginal pH and potassium hydroxide (KOH) tests were not performed on ED vaginal wet prep samples, so the ED diagnosis of BV was clinical, based on history and physical examination findings and laboratory data rather than incorporating all of Amsel’s criteria. Women were considered to have a vaginal coinfection if they met two or more of the following conditions: diagnosis of vulvovaginal candidiasis, diagnosis of BV, or positivity for *T. vaginalis*.

Patients were considered infected with chlamydia or gonorrhea on the basis of the results of a nucleic acid amplification test (NAAT). Patients were deemed infected with trichomonas if they tested positive on a NAAT or if the parasite was identified on urinalysis or a wet prep. Patients were only considered to be negative for *T. vaginalis* if they had negative NAAT results. Patients with certain International Classification of Diseases, Ninth Revision, and International Classification of Diseases, Tenth Revision, codes for the ED were considered to have BV (codes N76.0, O86.13, 616.10, or 646.6) and vulvovaginal candidiasis (codes B37.3, B37.4, B37.42, B37.49, 112.1, or 112.2). Patients were considered treated for *Neisseria gonorrhoeae* and *Chlamydia trachomatis* if they received ceftriaxone or cefixime plus azithromycin in the ED, or received doxycycline as a prescription. Patients with missing or erroneous data were not included in the analysis.

Statistical analysis

Categorical variables were summarized with frequency and percentage and analyzed with chi-square test. Continuous variables were summarized with mean (SD) and analyzed with Wilcoxon rank-sum test. Odds ratios (ORs) for continuous variables were calculated as the per-unit change in the regressor. Regression analysis was performed with adjustment for age and race. Analyses were performed with statistical software (JMP Pro 14; SAS Corp., Cary, NC, USA). All tests were two-sided, and statistical significance was set at P≤.05. We followed the STROBE (Strengthening the Reporting of Observational Studies in Epidemiology) reporting guidelines for this study.

## Results

Demographic characteristics

There were 17,184 patient encounters where a woman received a vaginal wet prep in the ED. Among these, the results of 16,484 women with complete vaginal wet prep data were analyzed (Table 1). Mean (SD) age was 28.7 (9.3) years. Most patients were Black/African American and single. Most patients did not have a primary care doctor, had a body mass index of 25 or higher, and were discharged from the ED. The patients had a mean (SD) emergency severity index (ESI) level of 3.2 (0.5) and a mean (SD) triage pain scale of 5.2 (3.7) (range, 0-10). Most patients were tested for *C. trachomatis* or *N. gonorrhoeae* with a NAAT, and 9.6% were positive for either or both infections. Overall, 1,802 patient encounters in the data set (10.9%) tested positive for *T. vaginalis*. This protozoan parasite was identified in 2.5% (266/10,542) of urinalyses, 8.4% (1,377/16,484) of vaginal wet preps, and 8.4% (406/4,821) of NAATs with some women having *T. vaginalis* being diagnosed on multiple tests. Only 29.2% (n=4,821) of women underwent NAAT testing for *T. vaginalis*. There were 71.0% (4,415/6,217) of women that were negative for *T. vaginalis *by NAAT.

**Table 1:** Baseline Characteristics of Patients Undergoing Vaginal Wet Preparation in the ED Abbreviations: BMI, body mass index; *C. trachomatis*; *Chlamydia trachomatis*; ED, emergency department; ESI, emergency severity index; N, number; NAAT, nucleic acid amplification test; *N. gonorrhoeae*, *Neisseria gonorrhoeae*; SD, standard deviation; *T. vaginalis, Trichomonas vaginalis*; vs, versus; y, year. a Values are presented as percentage (number) of patients unless specified otherwise. b Considered positive for *T. vaginalis* infection if seen on urinalysis or wet preparation or with positive NAAT. Considered negative for *T. vaginalis* only if negative on NAAT. Those who were negative for *T. vaginalis* on wet preparation but did not have NAAT were not included in the denominator.

Characteristic	Value^a^ (N=16,484)
Age, mean (SD), y	28.7 (9.3)
Race	
Black/African American	88.7 (14,557/16,414)
White	9.9 (1,629/16,414)
Asian	0.3 (53/16,414)
Other	1.1 (175/16,414)
Marital status	
Single	86.2 (14,214/16,482)
Married	9.3 (1,531/16,482)
Divorced	2.8 (457/16,482)
Separated	0.9 (140/16,482)
Life partner	0.1 (8/16,482)
Widowed	0.5 (89/16,482)
Unknown	0.3 (43/16,482)
Discharge from ED (vs admitted)	94.2 (15,524/16,484)
Primary care doctor (vs not)	17.5 (2,883/16,484)
ESI mean (SD) (range, 1-5)	3.2 (0.5) (n=15,747)
BMI <25 (vs ≥25)	32.9 (367/1,114)
Pain scale, mean (SD) (range, 0-10)	5.2 (3.7) (n=1,757)
Pregnancy (vs not)	21.9 (3,610/16,484)
Diagnosis of bacterial vaginosis	25.0 (4,124/16,484)
Diagnosis of candidiasis	3.8 (625/16,484)
Diagnosis of trichomoniasis	4.5 (732/16,484)
Underwent NAAT for T vaginalis	29.2 (4,821/16,484)
Positive for T vaginalis^b^	29.0 (1,802/6,217)
Underwent NAAT for N gonorrhoeae or C trachomatis, or both	97.2 (16,021/16,484)
Positive NAAT for N gonorrhoeae or C trachomatis, or both	9.6 (1,583/16,484)

Black/African American women were more likely to be diagnosed with BV (26.4%), vulvovaginal candidiasis (4.0%), and trichomoniasis (4.8%) than women who were not Black/African American (13.7%, 2.3%, and 1.9%, respectively) (P≤.002). Black/African American women also were more likely to be diagnosed with a vaginal coinfection (1.9%, n=270) compared with women who were not Black/African American (0.8%, n=14) (P<.001).

No statistically significant difference was observed in the ages between those diagnosed with BV (mean [SD], 28.8 [9.01] years) and those with no BV diagnosis (mean [SD], 28.7 [9.42] years) (P=.19). The same outcome was observed for patients with and without vulvovaginal candidiasis (mean [SD], 28.9 [10.5] years vs mean, 28.7 [9.28] years) (P=.29). The women diagnosed with trichomonas infection in the ED were older (mean [SD], 30.3 [9.79] years) than those without it (mean [SD], 28.6 [9.30] years) (P<.001). Women with vaginal coinfections were older (mean, 30.3 years [SD, 10.17]) than those without coinfection (mean, 28.7 years [SD, 9.31]) (P=.006).

Vaginal wet prep results on microscopy

The results of the vaginal wet prep findings are summarized in Table 2. The mean (SD) number of vaginal WBCs was 13.0/HPF (17.6/HPF). The following were present on vaginal wet prep: *T. vaginalis*, 8.4% (n=1,377); clue cells, 42.4% (n=6,988); yeast, 6.5% (n=1,065); clue cells and yeast, 2.3% (n=383); clue cells and *T. vaginalis*, 3.3% (n=547); and yeast and *T. vaginalis*, 0.4% (n=61). Clue cells, *T. vaginalis*, and yeast were all present from the same patient in only 0.2% (n=27) of all vaginal wet preps.

**Table 2:** Vaginal Wet Preparation Findings on Microscopy Abbreviations: HPF, high-power field; N, number; SD, standard deviation; *T. vaginalis*, *Trichomonas vaginalis*; WBC, white blood cell. a Values are presented as number (%) of vaginal wet preparations unless specified otherwise.

Result	Value^a ^(N=16,484)
WBC/HPF, mean (SD), No	13.0 (17.6)
+ Clue cells	6,988 (42.4)
+ Yeast	1,065 (6.5)
+ Trichomonas vaginalis	1,377 (8.4)
+ Clue cells and + yeast	383 (2.3)
+ Clue cells and + T vaginalis	547 (3.3)
+ Yeast and T vaginalis	61 (0.4)
+ Clue cells and + T vaginalis and + yeast	27 (0.2)

Association between vaginal wet prep results and ED diagnoses

Patients were diagnosed with the following: BV, 25.0% (n=4,124); vulvovaginal candidiasis, 3.8% (n=625); trichomoniasis, 4.5% (n=732); BV and vulvovaginal candidiasis, 0.8% (n=136); vulvovaginal candidiasis and trichomoniasis, 0.1% (n=20); BV and trichomoniasis, 0.9% (n=150); and trichomoniasis, BV, and vulvovaginal candidiasis, 0.1% (n=10) (Table 3). Overall, 1.9% (316/16,484) of women undergoing vaginal wet prep were diagnosed with a vaginal coinfection.

**Table 3:** Diagnoses and the Associated Vaginal Wet Preparation Results Abbreviation: N, number; SD, standard deviation; *T. vaginalis*, *Trichomonas vaginalis*; WBC, white blood cell. a Values are presented as number (%) of patients unless specified otherwise. b Considered positive for *T. vaginalis* when seen on urinalysis or wet preparation or with a positive NAAT. Considered negative for *T. vaginalis* only when a negative NAAT. Those who tested negative for *T. vaginalis* with wet preparation but who did not have NAAT were not included in the denominator.

	Vaginal wet preparation result^a ^(N=16,484)					
Diagnosis	WBCs, mean (SD)	Clue cells	Yeast	T vaginalis	+ T vaginalis^b^	Overall %
Vulvovaginal candidiasis (N=625)	17.8 (21.3)	178 (28.5)	455 (72.8)	23 (3.7)	36 (5.8)	3.8
Bacterial vaginosis (N=4,124)	15.4 (17.0)	3,663 (88.8)	232 (5.6)	222 (5.4)	298 (7.2)	25.0
T vaginalis infection (N=737)	21.7 (23.7)	286 (39.1)	39 (5.3)	689 (94.1)	713 (99.6)	4.5
Vulvovaginal candidiasis and bacterial vaginosis (N=136)	17.5 (20.3)	121 (89.0)	104 (76.5)	11 (8.1)	15 (11.0)	0.8
Vulvovaginal candidiasis and T vaginalis infection (N=20)	18.6 (17.6)	11 (55.0)	20 (100)	17 (85)	18 (100)	0.1
Bacterial vaginosis and T vaginalis infection (N=150)	19.7 (22.1)	144 (96.0)	14 (9.3)	141 (94.0)	146 (99.3)	0.9
Vulvovaginal candidiasis, bacterial vaginosis, and T vaginalis infection (N=10)	14.9 (12.2)	10 (100.0)	10 (100.0)	9 (90.0)	10 (100.0)	0.1

Vaginal WBCs

Mean vaginal WBC counts were higher for patients whose vaginal wet prep showed clue cells, yeast, and *T. vaginalis* (P≤.001 for all) (Table 4). Additionally, mean vaginal WBC counts on wet prep were higher for those with vaginal coinfections than those without a coinfection (P≤.001).

**Table 4:** Vaginal WBCs on Wet Preparation Abbreviation: HPF, high-power field; N, number; SD, standard deviation; *T. vaginalis*, *Trichomonas vaginalis*; WBC, white blood cell.

Result on vaginal wet preparation	Vaginal WBC/HPF, mean (SD), N.; N. of wet preparations
Clue cells (+)	13.3 (17.1); 6,988
Clue cells (-)	12.7 (18.0); 9,496
Yeast (+)	19.8 (21.8); 1,065
Yeast (-)	12.5 (12.5); 15,419
T vaginalis (+)	21.6 (23.2); 1,377
T vaginalis (-)	12.2 (16.8); 15,107
Clue cells and yeast (+)	17.4 (18.9); 383
Clue cells and yeast (-)	12.8 (17.6): 16,101
Clue cells and T vaginalis (+)	20.2 (22.1); 547
Clue cells and T vaginalis (-)	12.7 (17.4); 15,937
Yeast and T vaginalis (+)	20.6 (22.3); 581
Yeast and T vaginalis (-)	12.7 (17.4); 15,903
Clue cells, yeast, and T vaginalis (+)	26.9 (26.4); 27
Clue cells, yeast, and T vaginalis (-)	12.9 (17.6); 16,457

Vaginal coinfection compared with no coinfection

Older age, Black/African American race, discharge from the ED, having a primary care doctor, a higher ESI score, higher vaginal WBC count, higher urine WBC count, more urine bacteria, and higher leukocyte esterase level were associated with higher odds of having a vaginal coinfection on both univariable and regression analyses compared with those with no coinfection (P≤.04 for all) (Table 5). On both univariable and regression analysis patients with a vaginal coinfection were not more likely to be infected with *C. trachomatis* and/or *N. gonorrhoeae* or have ≥10 urine red blood cells on urinalysis. More women with a vaginal coinfection were treated for gonorrhea and chlamydia in the ED (P<.001).

**Table 5:** Associations With a Diagnosis of Vaginal Coinfection Abbreviations: CI, confidence interval; ED, emergency department; ESI, emergency severity index; HPF, high-power field; OR, odds ratio; RBC, red blood cell; *T. vaginalis*, *Trichomonas vaginalis*; vs, versus; WBC, white blood cell; y, year. a Presented as percentage and number of patients or mean (SD) and number of patients. b Considered positive for *T. vaginalis* if seen on urinalysis or wet preparation, or had a positive NAAT. Considered negative for *T. vaginalis* only if negative by NAAT. Those who were negative for *T. vaginalis* by wet preparation but who did not have a NAAT were not included in the denominator.

Characteristic	+ Vaginal coinfection^a^	- Vaginal coinfection^a^	OR (95% CI)	P value	Adjusted OR (95% CI)	Adjusted P value
Age, y	30.3 (10.2); 286	28.7 (9.3); 16,198	NA	.008	1.02 (1.01-1.03)	.001
Black/African American race (vs not)	95.1 (270/284)	88.6 (14,287/16,130)	2.49 (1.45-4.27)	< .001	2.57 (1.50-4.40)	< .001
Married/life partner (vs other)	8.0 (23/286)	9.4 (1,516/16,153)	0.84 (0.55-1.30)	.54	0.87 (0.56-1.35)	.53
Admitted from ED (vs admit)	2.8% (8/286)	5.9% (952/16,198)	.46 (.23-.93)	.03	.47 (.23-.96)	.04
Primary care doctor (vs not)	25.2 (72/286)	17.4 (2,811/16,198)	1.60 (1.22-2.10)	< .001	1.61 (1.23-2.13)	< .001
ESI (range, 1-5)	3.3 (0.5); 261	3.2 (0.5); 15,486	NA	< .001	1.63 (1.29-2.07)	< .001
Triage pain scale (range, 0-10)	5.2 (4.0); 33	5.2 (3.7); 1,724	NA	.97	1.00 (0.91-1.10)	.93
Wet preparation WBC, cells/HPF	18.9 (21.5); 286	12.8 (17.5); 16,198	NA	< .001	1.01 (1.01-1.02)	< .001
Wet preparation +clue cells	89.5 (256/286)	41.6 (6,732/16,198)	12.0 (8.21-17.54)	< .001	11.86 (8.10-17.37)	< .001
Wet preparation +yeast	41.3 (118/286)	5.9 (947/16,198)	11.31 (8.86-14.44)	< .001	11.44 (8.94-14.63)	< .001
Wet preparation + T vaginalis	52.8 (151/286)	7.6 (1,226/16,198)	13.66 (10.75-17.35)	< .001	12.87 (10.10-16.38)	< .001
Positive for T vaginalis^b^	87.5 (161/184)	27.2 (1,641/6,033)	18.7 (12.1-29.1)	< .001	17.96 (11.53-27.98)	< .001
Infection with Neisseria gonorrhoeae, Chlamydia trachomatis, or both	11.7 (33/281)	9.9 (1,556/15,782)	1.22 (0.84-1.76)	.31	1.30 (0.89-1.91)	.17
Urine WBCs, ≥10 cells/HPF	142 (61.7); 230	4197 (40.6); 10,346	NA	< .001	2.37 (1.81-3.11)	< .001
Urine RBCs, ≥10 cells/HPF	65 (28.4); 229	3064 (29.6); 10,344	NA	.71	0.95 (0.71-1.27)	.71
Urine bacteria (0, 1+, 2+, 3+, 4+)	1.2 (1.2); 231	1.0 (1.1); 10,345	NA	.001	1.19 (1.06-1.33)	.002
Urine leukocyte esterase (0, 1+, 2+, 3+, or 4+)	1.8 (1.3); 260	0.8 (1.1); 14,339	NA	< .001	1.88 (1.70-2.07)	< .001
Positive urine nitrite (vs negative)	4.9 (13/263)	3.7 (531/14,370)	1.4 (0.8-2.4)	.32	1.35 (0.76-2.37)	.30
Urine pH (range, 5-9)	6.0 (0.9); 263	6.0 (0.9); 14,374	NA	.97	1.02 (0.89-1.17)	.74
Metronidazole given in ED or prescription (vs not)	87.4 (250/286)	39.6 (6,412/16,198)	10.6 (7.5-15.1)	< .001	10.56 (7.39-15.09)	< .001
Treated for gonorrhea/chlamydia in ED (vs not)	35.0 (100/286)	18.2 (2,954/16,198)	2.4 (1.9-3.1)	< .001	2.37 (1.85-3.04)	< .001

## Discussion

Vaginal coinfections of candidiasis, trichomonas, and BV are uncommon and only occurred in 1.9% (316/16,484) of women undergoing vaginal wet prep in the ED. The most common causes of vaginal coinfections were BV and trichomoniasis, vulvovaginal candidiasis and trichomoniasis, and vulvovaginal candidiasis and BV. Cases of vulvovaginal candidiasis, trichomoniasis, and BV were rare. Vaginal coinfections were significantly associated with older age, Black/African American race, having a primary care doctor, having a higher ESI level, treatment of gonorrhea and chlamydia, discharge from the ED, a greater vaginal WBC count, and on urinalysis having greater values of WBCs, bacteria, and leukocyte esterase. Some findings were in concordance with what has been published previously, such as trichomonas occurrence in older women; that Black/African American women were more likely to be diagnosed with BV, vulvovaginal candidiasis, and trichomoniasis; and that BV and trichomonas are the most common causes of vaginal coinfection [5,14,18-24].

The most common cause of vaginitis diagnosed in the ED was BV, followed in order of frequency by *T. vaginalis* and vulvovaginal candidiasis. The prevalence of these diseases in the ED is different than what has been reported in outpatient clinics, where vulvovaginal candidiasis has been reported with greater frequency [7,11]. Additionally, previous studies have shown that both *T. vaginalis* and BV are associated with *N. gonorrhoeae* and *C. trachomatis* infection and that *T. vaginalis* can be associated with BV [19,25-27]. Our data did not show that vaginal coinfections were associated with *N. gonorrhoeae* or *C. trachomatis*, or both.

Limitations

The data set contained patient encounters and some patients presented to the ED multiple times. The ED patients in our data set were likely presenting with genitourinary complaint in order to receive a vaginal wet prep in the ED. The data set lacked racial diversity, and all data came from hospitals in northeast Ohio. Since only ED patient encounters were examined our results are not likely to be generalizable to all women or those being evaluated in outpatient clinics. Only the three most common vaginal infections in the ED were examined in this study; other causes of vaginitis, such as aerobic vaginitis and cytolytic vaginosis, are not typically diagnosed in the ED [5,28]. For some patients for whom coinfections were identified, one pathogen may not have caused any vaginal symptoms. For instance, 20% of asymptomatic women had culturable yeast from the vagina, and the majority of women with trichomonas infection may be asymptomatic [5,12]. Neither vaginal pH measurements nor the whiff test (ie, potassium hydroxide (KOH) added to the vaginal discharge) was performed in the ED or on ED samples, thus preventing the calculation of Amsel criteria for BV. Yet, the presence of two of the four Amsel criteria (eg, a gray-white thin or watery discharge plus clue cells) may perform as well as three of four criteria for the diagnosis of BV [5,29]. The Nugent score for diagnosing BV is rarely used clinically and was not used in the ED for the BV diagnosis [5]. Neither direct probe assays nor NAATs were used to aid in the BV diagnosis [5]. The use of molecular tests would likely have increased the number of positive single infections and coinfections in our data set [30].

## Conclusions

Vaginal coinfections with *T. vaginalis*, BV, and candidiasis are infrequent, occurring in only 1.9% of women undergoing wet prep in the ED. The most common vaginal coinfections were BV and trichomonas, candidiasis and BV, candidiasis and trichomonas, and BV, candidiasis, and trichomonas. Vaginal coinfections were associated with older age, Black/African American race, having a primary care doctor, and not being married or not having a life partner. Women with a vaginal coinfection were not more likely to be infected with *N. gonorrhoeae* or *C. trachomatis*, or both, but were more likely to be treated for gonorrhea and chlamydia in the ED. Vaginal coinfections were found to be associated with a greater number of vaginal WBCs, urine WBCs, and urine bacteria and with greater urine leukocyte esterase values.
